# 1118. Population Pharmacokinetics of Contezolid Acefosamil and Contezolid – Rationale for a Safe and Effective Loading Dose Regimen

**DOI:** 10.1093/ofid/ofab466.1311

**Published:** 2021-12-04

**Authors:** Jürgen B Bulitta, Barry Hafkin, Edward Fang

**Affiliations:** 1 University of Florida, Orlando, Florida; 2 MicuRx Pharmaceuticals / Consultant, Austin, Texas; 3 MicuRx Pharmaceuticals, San Carlos, California

## Abstract

**Background:**

Contezolid (CZD) is a novel oral oxazolidnone with comparable activity and potentially improved safety compared to current oxazolidinones. The intravenous (IV) double prodrug contezolid acefosamil (CZDa) is converted via MRX-1352 to active CZD. CZDa paired with CZD holds promise as a safe and effective treatment for serious Gram-positive infections such as those caused by methicillin-resistant *Staphylococcus aureus*. Sequential therapy with CZDa IV followed by CZD oral (PO) offers flexible treatment options in hospital and outpatient settings for conditions such as diabetic foot infections. We aimed to design a CZDa/CZD dosage regimen leveraging population pharmacokinetic modeling (PopPK).

**Methods:**

PopPK simultaneously fit data from 184 adult subjects. These were 1) plasma concentrations (by LC-MS/MS) of MRX-1352, CZD, and its metabolite MRX-1320 from 66 healthy subjects receiving CZDa (150-2400 mg IV) for up to 10 days, 2) CZD and MRX-1320 concentrations from 44 healthy subjects receiving single CZD PO doses of 400, 800, or 1200 mg with and without food or multiple doses Q12h for up to 28 days, and 3) CZD concentrations from 74 Phase 2 patients receiving CZD 800 mg PO Q12h. PopPK and Monte Carlo simulations were used to optimize CZD exposures.

**Results:**

CZDa was rapidly converted to MRX-1352, which was converted less rapidly to CZD. CZD was well absorbed and food enhanced its bioavailability. For CZD 800 mg PO with food, apparent total clearance of CZD was 13.1 L/h (22% coefficient of variation) in healthy subjects and 14.5 L/h (53% CV) in patients. The apparent volume of distribution at steady-state was 20.5 L. A loading dose of CZDa 2000 mg IV, then CZDa 1000 mg IV Q12h, and followed by CZD 800 mg PO Q12h achieved areas under the curve (AUC) between 75 and 100 mg*h/L (medians; Figure) on all study days. Compared to CZD AUCs, the MRX-1352 AUCs during IV dosing were higher. While the median MRX-1320 AUCs were lower (18 to 48 mg*h/L), some accumulation was predicted in ~5% of subjects.

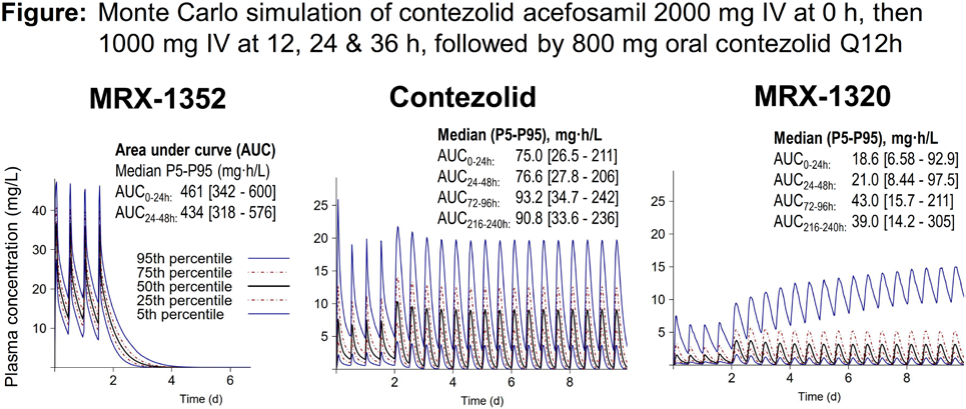

**Conclusion:**

A loading dose of CZDa 2000 mg IV followed by either CZDa 1000 mg IV or CZD 800 mg PO Q12h was predicted to reliably achieve efficacious CZD exposures on day 1 and maintain those exposures throughout therapy. This regimen will be evaluated in Phase 3 studies in complicated skin infections and diabetic foot infections.

**Disclosures:**

**Jürgen B. Bulitta, PhD**, **MicuRx Pharmaceuticals, Inc.** (Consultant) **Barry HAFKIN, MD**, **MicuRx Pharmaceuticals Inc.** (Consultant)

